# The ubiquity of uncertainty: a scoping review on how undergraduate health professions’ students engage with uncertainty

**DOI:** 10.1007/s10459-021-10028-z

**Published:** 2021-03-01

**Authors:** Jenny Moffett, Jennifer Hammond, Paul Murphy, Teresa Pawlikowska

**Affiliations:** 1 RCSI Health Professions’ Education Centre, 123 St Stephen’s Green, Dublin, Ireland; 2 School of Veterinary Medicine, College of Medical, Veterinary and Life Sciences, University of Glasgow, Glasgow, UK

**Keywords:** Ambiguity, Learning, Teaching, Uncertainty, Undergraduate

## Abstract

Although the evidence base around uncertainty and education has expanded in recent years, a lack of clarity around conceptual terms and a heterogeneity of study designs means that this landscape remains indistinct. This scoping review explores how undergraduate health professions' students learn to engage with uncertainty related to their academic practice. To our knowledge, this is the first scoping review which examines teaching and learning related to uncertainty across multiple health professions. The scoping review is underpinned by the five-stage framework of (Arksey and O'Malley in Scoping studies: Towards a methodological framework International Journal of Social Research Methodology 8(1) 19-32, 2005). We searched MEDLINE, Embase, PsychINFO, ISI Web of Science, and CINAHL and hand-searched selected health professions’ education journals. The search strategy yielded a total of 5,017 articles, of which 97 were included in the final review. Four major themes were identified: “Learners’ interactions with uncertainty”; “Factors that influence learner experiences”; “Educational outcomes”; and, “Teaching and learning approaches”. Our findings highlight that uncertainty is a ubiquitous concern in health professions’ education, with students experiencing different forms of uncertainty at many stages of their training. These experiences are influenced by both individual and system-related factors. Formal teaching strategies that directly support learning around uncertainty were infrequent, and included arts-based teaching, and clinical case presentations. Students also met with uncertainty indirectly through problem-based learning, clinical teaching, humanities teaching, simulation, team-based learning, small group learning, tactical games, online discussion of anatomy topics, and virtual patients. Reflection and reflective practice are also mentioned as strategies within the literature.

## Introduction

Health professionals regularly encounter uncertainty in their work, experiencing “a subjective perception of not knowing what to think or what to do” (Sommers and Launer 2014). Indeed, it is accepted that uncertainty is “normal, understandable, and to be expected in professional practice” (Coles 2013). When confronted with complex or ambiguous situations, individuals react in different ways, often framed in terms of their cognitive, emotional and behavioural responses (Mushtaq et al. 2011; Strout et al. 2018). These differences, and the capacity of health professionals to manage uncertainty overall, are often referred to as “uncertainty tolerance.” Studies, largely in medicine, have found that professionals’ capacity to manage uncertainty is important with respect to their career choices (Merrill et al. 1994; Cranley et al. 2012; Caulfield et al. 2014), attitudes to patients (Merrill et al. 1994; Wayne et al. 2011), clinical decision-making skills (Merrill et al. 1994; Strout et al. 2018), and exposure to work-related stress (Logan and Scott 1996; Bovier and Perneger 2007; Lally and Cantillon 2014; Iannello et al. 2017; Simpkin et al. 2018). Furthermore, a professional’s capacity to work with uncertainty has been linked to positive outcomes for others, e.g., greater patient satisfaction (Johnson et al. 1988; Gordon et al. 2000) and decreased medical errors (Light 1979; Fielding 1999). A recent review by Strout and colleagues (2018) highlighted a strong, consistent association between health professionals’ uncertainty tolerance, and their patients’ emotional well-being. This growing evidence base has encouraged the addition of uncertainty management competences to many regulatory professional frameworks (AMRC 2009; Benson et al. 2015; GMC 2018; RCVS 2018).

Considering this increasing research interest, relatively less attention has been paid to how health professions’ learners build this capacity to work with uncertainty. Existing studies point to a long-standing balancing act between the overarching human preference for certainty and the uncertain nature of real-world patient care (Fox 1957; Atkinson 1984; Katz 1984; Beresford 1991; Han et al. 2011; Simpkin and Schwartzstein 2016). Authors suggest that we have consistently failed to bridge the gap between the two, labeling training for uncertainty as medical education’s “most elusive ideal” (Ludmerer 1999). This contributes to an educational climate which “rewards those who give correct answers, and often denigrates learners who admit uncertainty" (Wray and Loo 2015).

It has also been argued that health professions’ education may have come adrift with regards to preparing learners for the “messiness and unpredictability” of professional practice (Wilkinson 2017). Wear (2009) hypothesises that the “rapid shift... to a technology-driven, competency-oriented environment” may mean that learners have less opportunity to develop “responsiveness to an evolving human situation in a clinical context.” Indeed, could our modern curricula, “bloated with required lectures and courses, with insufficient time for independent thought and elective study”, lie at the heart of the problem? (Ludmerer 1999).

Authors have recommended specific ways to facilitate learning around uncertainty, from humanities teaching, small group approaches, and simulation (Hazel et al. 2013; Bleakley and Marshall 2013; Wald et al. 2015; Ofri 2017; White and Williams 2017; Tonelli and Upshur 2019), through to faculty development (Domen 2016; George and Lowe 2019). Taken as a whole, however, little is known about how health professions’ programmes “intentionally and systematically” teach students to manage uncertainty (Ledford et al. 2015). This leaves educators in a position where they are asked to support learning around uncertainty, but with little clear advice on how best to do this (Cooke and Lemay 2017; Ofri 2017; White and Williams 2017).

Although the evidence base around uncertainty and education has expanded in recent years, a lack of clarity around conceptual terms and a heterogeneity of study designs means that this landscape remains indistinct, replete with “fuzzy” boundaries (Grenier et al. 2005; Hillen et al. 2017; Strout et al. 2018). This hinders educators’ ability to prepare health professions’ learners to work with the uncertainty inherent in their day-to-day work. The authors considered that the existing literature could be usefully “mapped”, making what we know so far in relation to uncertainty and education more accessible. Our aim was to explore how learners from a range of different health professions begin to learn about uncertainty within the context of their education. As our interest extended across multiple professions, we decided to focus on findings related to undergraduate health professions’ learners as these may be more broadly comparable. We chose a scoping review approach to provide an overview of this emergent evidence base. This was considered an appropriate methodology which could help us unravel what research exists, and what characteristics or factors are important when considering uncertainty in health professions’ education (Munn et al. 2018). To our knowledge, this is the first scoping review which examines teaching and learning related to uncertainty across multiple health professions.

## Methods

We followed the scoping review framework described by Arksey and Malley (2005), and incorporated guidance by Peters and colleagues (2015). The five steps of the framework were: (1) identifying the research question, (2) identifying relevant studies, (3) selection of relevant studies, (4) charting the data, and (5) collating, summarising and reporting the results. In addition, we used the Preferred Reporting Items for Systematic reviews and Meta-Analyses extension for Scoping Reviews (PRISMA-ScR) to guide reporting of the study (Tricco et al. 2018) (Appendix 1).

**Fig. 1:**
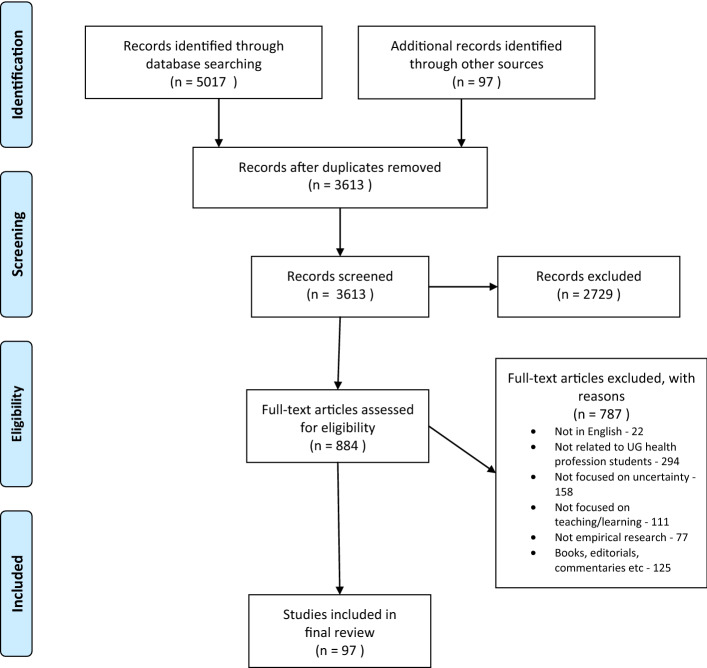
PRISMA ScR
- *Stage 4*Charting the data
- Data extraction followed an iterative process, and a template was used to extract the following information: publication details (authors, publishing year, title of journal and paper), country of origin, study design, study population, research outcome(s), type and description of intervention, if any, as well as key findings that related to the research question. We used a combination of Microsoft Excel and Forms (Microsoft, USA) to extract the data, with the characteristics of the full-text articles extracted independently by JM and JH. Studies were excluded at this stage if they did not meet eligibility criteria. Discrepancies were solved through re-reading and discussing studies in consultation with TP.
- *Stage 5* Collating, summarising and reporting the results
- We used a narrative approach to thematically synthesise the data (Braun and Clarke 2013); JM and JH identified initial themes within the studies. These were shared, mapped and discussed iteratively, which helped visualisation of the data and recognition of connections between themes. The third researcher (TP) addressed any discrepancies to ensure consensus was reached.

- *Stage 1* Identifying the review question
- Following a pilot search, we decided to focus on how undergraduate health professions’ learners both experience and respond to uncertain situations. The final wording for the research question was: “How do undergraduate health professions' students learn to engage with uncertainty related to their academic practice?" We adopted a broad definition which framed uncertainty as a “subjective perception of ignorance that is experienced by health professionals in differing ways and degrees, motivates action, and elicits a variety of psychological responses” (adapted from Han and colleagues, 2011). Our focus on undergraduate learners took into consideration the different models and approaches to health professions’ education which exist (Wijnen-Meijer et al. 2013). Thus, we were interested in studies which related to students enrolled on health professions-specific, college-level courses which would lead to registration to practise in their chosen profession. Finally, we chose the verb “engage”, so as to capture both learners’ experiences of, and responses to, uncertainty, as these were both deemed of interest.
- *Stage 2* Identifying relevant studies
- We devised the search strategy in consultation with an academic librarian through an iterative process using both keywords and Medical Subject Headings (MeSH) terms. Due to conceptual overlap between uncertainty and ambiguity, which was evident in the literature and within our pilot search, both terms were included in the search (Grenier et al. 2005; Rosen et al. 2014; Hillen et al. 2017).
- We searched MEDLINE, Embase, PsychINFO, ISI Web of Science, and CINAHL (sample strategy included as Appendix 2). In addition, we carried out a hand search of 14 health professions’ education journals (Appendix 3), and completed a backward citation search of all articles which met the review criteria. We limited all strands of the search to studies published from January 1, 1950 until September 14, 2020.
- *Stage 3* Selection of relevant studies
- We used EndNote X7.8 (Thomson Reuters, USA) to import and organise the citations of articles yielded from the search strategy. Initially, articles were grouped according to their source, and duplicate citations were removed. Researchers JM and JH independently reviewed a group of 50 studies in tranches to pilot the initial eligibility criteria, and make any necessary refinements. Studies were included in this review on the basis of an agreed set of inclusion and exclusion criteria (Table 1). JM and JH independently screened titles and abstracts of the studies to identify those eligible for full-text review. A third researcher (TP) was consulted on disagreements until consensus was attained (Fig. 1). All studies deemed relevant were submitted for full-text screening. Again JM and JH independently screened studies, with TP facilitating consensus.

**Table 1:** The ubiquity of uncertainty: a scoping review on how undergraduate health professions’ students engage with uncertainty: Inclusion and exclusion criteria

Inclusion criteria	Exclusion criteria
Articles were included in this scoping review if they: Were published in English Related to undergraduate health professions’ students (limited to medicine, nursing, midwifery, dentistry, veterinary medicine, physical therapy and/or physiotherapy, pharmacy students) Focused on uncertainty in the context of the individual’s professional practice Focused on teaching and learning as reported by student rather than other stakeholders Described empirical research (i.e., represented a peer‐reviewed article with overt data collection)	Articles were excluded from this scoping review if they: Related to postgraduate education or continuing professional development Focused on teaching and learning from the perspective of the educator or patient, or from broader paradigms e.g., educational development Were books, commentaries, conference abstracts, editorials, letters, opinion papers, or unpublished theses

## Results

### Characteristics of included studies

The search strategy yielded a total of 5,017 articles, of which 97 articles were included in the final review (Fig. 1).

Of these studies, half had been published within the last five years (50%, n = 48), with the USA the most frequently reported location (35%, n = 34), followed by the UK (20%, n = 19), and Canada (11%, n = 11). Studies described both uniprofessional (90%, n = 87) and multiprofessional (10%, n = 10) student cohorts. The most commonly represented students were medical (65%, n = 63), followed by nursing (25%, n = 24). Studies were more likely to describe qualitative research (57%, n = 55), than quantitative (32%, n = 31), or mixed method approaches (11%, n = 11). A summary of the final study characteristics is presented in Table 2.

**Table 2:** The ubiquity of uncertainty: a scoping review on how undergraduate health professions’ students engage with uncertainty: summary of main characteristics of the final studies

Authors	Year	Country	Discipline	Study population (n)	Study population	Research design	Methods	Relevant teaching/ learning strategies
Ali, MA et al.	2017	Canada	Multiprofessional	6	Nursing students; Students from other professions	Qualitative	Interviews; Focus groups	Field placements/Clinical internships
Al-Kloub, M et al	2019	Jordan	Uniprofessional	130	Third-year medical students	Mixed methods	Questionnaire (PBL Evaluation Questionnaire); Observation (daily logs)	Problem-based learning
Balentine, CJ et al	2010	USA	Uniprofessional	236	Medical students	Quantitative	Questionnaire (Patient Provider Orientation Scale, Physician Reaction to Uncertainty)	N/A
Bassett, AM et al	2015	UK	Uniprofessional	26	Nursing students (mental health)	Qualitative	Interviews	Clinical setting
Bentwich, ME et al	2017	Israel	Uniprofessional	67	First-year medical students	Quantitative	Questionnaire (open and closed questions)	Visual thinking strategies (arts-based learning)
Biley, F & Smith, K	1999	UK	Uniprofessional	17	Nursing students	Qualitative	Interviews; Observation data	Problem-based learning
Bing-You, R	1991	USA	Uniprofessional	47	Medical students	Quantitative	Questionnaire (Scott’s Value Scale, Webster’s Authoritarian Scale)	Clinical clerkships
Bintley, HL et al	2019	UK	Uniprofessional	40	Medical students	Mixed methods	Examination performance; Focus groups	Simulation-based learning
Brondani, M & Donnelly, L	2020	Canada	Uniprofessional	115	Dentistry students	Qualitative	Observation data (reflective writing)	Reflection
Carr, S et al	2001	UK	Uniprofessional	nc	Nursing students; Educators	Qualitative	Interviews; Focus Groups; Observation data (practice narratives)	Practice experience of nursing in a clinical community context
Chan, EA & Nyback, MH	2015	Finland / China (Hong Kong)	Uniprofessional	20	First-year nursing students	Qualitative	Observation data (student projects, reflective writing)	Technology-enhanced learning (online cultural competence course)
Curtis, K	2014	UK	Uniprofessional	19	Nursing students	Qualitative	Interviews	N/A
Curtis, K et al	2012	UK	Uniprofessional	19	Nursing students; nb data from health service staff and patients, and educators, used to contextualise findings	Qualitative	Interviews	N/A
DeForge, BR & Sobal, J	1989	USA	Uniprofessional	609	First-year medical students	Quantitative	Questionnaire (Budner’s Intolerance of Ambiguity Scale)	N/A
Dodgson, JE et al	2018	Japan / USA	Multiprofessional	13	Nursing students	Qualitative	Interviews	N/A
Drummond, I et al	2016	UK	Uniprofessional	28	Final-year medical students	Qualitative	Focus groups	Tactical decision games
Duvivier, R et al	2014	Netherlands	Uniprofessional	32	Fourth-year medical students	Qualitative	Focus groups	Clinical clerkships
Eley, DS et al	2017	Australia	Uniprofessional	797	Medical students	Quantitative	Questionnaire (The Multiple Stimulus Types Ambiguity Tolerance Scale-II; The Resilience Scale; Frost Multidimensional Perfectionism Scale)	Clinical rural immersion programs
Evans, L et al	2012	USA	Uniprofessional	89	Medical students	Quantitative	Questionnaire (Physicians’ Belief Scale; Physicians’ Reactions to Uncertainty Scale)	Early clinical experience course and continuity clinical experience course
Fagundes, ED et al	2020	Brazil	Uniprofessional	60	Medical students	Quantitative	Observation data (audio-recorded case presentations)	SNAPPS; One-minute preceptor; Case presentations; Clinical clerkship
Fernandez, N et al	2016	Canada	Uniprofessional	404	Medical students	Qualitative	Questionnaire (open questions)	Learning-by-Concordance approach
Finnerty, G & Pope, R	2005	UK	Uniprofessional	3	Midwifery students	Qualitative	Observation data (audio-diaries)	Non-formal learning in the clinical setting
Framback, JM et al	2012	Netherlands	Uniprofessional	nc	Medical students; Educators	Qualitative	Interviews; Observation data (PBL tutorials)	Problem-based learning
Friary, P et al	2018	New Zealand	Multiprofessional	22	Physiotherapy students; Other students; Educators; Patients	Qualitative	Interviews; Focus groups	Interprofessional education
Ganesh, A & Ganesh G	2010	India	Uniprofessional	16	Final-year medical students	Qualitative	Observation data (written diaries)	Clinical setting
Gärtner, J et al	2020	Germany	Uniprofessional	67	Medical students	Qualitative	Observation data (video-recorded case presentations)	Simulation-based learning
Gaufberg, E et al	2018	USA	Uniprofessional	585	Medical students	Quantitative	Questionnaire (Jefferson Scale of Empathy; Patient- Practitioner Orientation Scale; Budner’s Tolerance of Ambiguity Scale; Ways of Coping Questionnaire–22 Item; Medical School Learning Environment Survey	Humanities activities
Geller, G et al	1990	USA	Uniprofessional	386	Medical students	Quantitative	Questionnaire (modified version of Budner’s Intolerance of Ambiguity)	N/A
Gibson, KR et al	2014	UK	Uniprofessional	183	Final-year medical students; Educators	Quantitative	Questionnaire (student and tutor versions); Attendance; Examination performance	A junior doctor-led prescribing tutorial programme
Gonzalo, JD et al	2020	USA	Uniprofessional	710	Medical students	Qualitative	Questionnaire (open questions)	Health systems science
Gormley, GJ & Fenwick, T	2016	UK	Uniprofessional	8	Fourth-year medical students	Qualitative	Interviews; Observation data (video footage)	Ward-based simulation teaching activity
Gowda, D et al	2018	USA	Multiprofessional	35,44,18	First-year medical students	Mixed methods	Questionnaire open and closed questions (Groningen Reflection Ability Scale, modified version of Budner’s Tolerance for Ambiguity scale, Best Intentions Questionnaire); Focus groups; Written narrative evaluations	Museum-based art course
Groot, F et al	2020	Netherlands	Uniprofessional	11	Medical students	Qualitative	Interviews	Simulation-based learning
Han, PKJ et al	2014	USA	Uniprofessional	28	Second-year medical students	Quantitative	Questionnaire (closed questions); Observation data (SP Risk Communication Process, Risk Communication Content)	Risk communication curriculum
Han, PKJ et al	2015	USA	Uniprofessional	58	Medical students	Quantitative	Questionnaire (Tolerance for Ambiguity, Pearson Risk Attitude, Ambiguity Aversion in Medicine)	N/A
Hancock, J et al	2017	UK	Multiprofessional	525	Medical students; Veterinary students	Quantitative	Questionnaire (Tolerance of Ambiguity of Medical Students and Doctors Scale, Tolerance of Ambiguity of Veterinary Students Scale)	N/A
Handwerker, SM	2018	USA	Uniprofessional	11	Nursing students	Qualitative	Interviews	N/A
Hayward, J et al	2016	Canada	Uniprofessional	301	Second-year medical students	Qualitative	Observation data (narrative response of student feedback on patient cases)	Virtual patients; Simulation; Case-based learning
Hazel, SJ et al	2013	Australia	Uniprofessional	264	First-year veterinary students; Other students	Mixed methods	Questionnaire open and closed questions; Student group marks	Team-based learning
He, B et al	2019	USA	Uniprofessional	65	Medical students	Qualitative	Questionnaire (open questions)	Arts-based learning
Helmich, E et al	2018	Netherlands/ Canada	Uniprofessional	29	Medical students; Doctors	Qualitative	Interviews	N/A
Huijer, M et al	2000	Netherlands	Uniprofessional	nc	Medical students	Quantitative	Case reports	Clinical setting
Ion, R et al	2015	UK	Uniprofessional	13	Final-year (adult and mental health) nursing students	Qualitative	Interviews	N/A
Ironside, PM	2003	USA	Uniprofessional	33	Nursing students; Educators	Qualitative	Interviews	N/A
Ironside, PM et al	2009	USA	Uniprofessional	413, 67	Final-year nursing students	Quantitative	Questionnaire (Multiple Stimulus Types Ambiguity Tolerance Scale-I, investigator-developed patient safety instrument)	Multiple- patient simulation experiences
Johnsen, H	2016	Denmark	Multiprofessional	71	Midwifery students; Physiotherapist students; Other students	Qualitative	Questionnaire, open ended questions; Focus groups	Technology enhanced learning: student projects
Jowsey, T et al	2020	New Zealand	Multiprofessional	115	Medical students; Pharmacy students, Nursing students; Other students	Qualitative	Observation data (participant observation, field notes, interviews, photography and observational ethnographic film)	Inter-professional learning; Simulation-based learning; Reflection
Kashbour, WA et al	2019	UK	Uniprofessional	28	Dentistry students	Qualitative	Focus groups	Clinical setting; Early clinical training
Klugman, CM et al	2011	USA	Multiprofessional	32	Medical students; Nursing students	Mixed methods	Questionnaire (Budner’s Tolerance of Ambiguity Scale, The Communication Skills Attitudes Scale); Texts (free responses to art and patient images)	Art rounds program/ visual thinking strategies
Koufidis, C et al	2020	Sweden	Uniprofessional	23	Medical students	Qualitative	Interviews; Observation data (participant observations, field interviews)	Clinical teaching
Kristiansson, MH et al	2014	Sweden	Uniprofessional	35	Medical students	Qualitative	Observation data (written reflections)	Reflective writing
Krupat, E et al	2011	USA	Uniprofessional	35	Third-year medical students	Mixed methods	Observation data (written reflections)	Clerkship/ Clinical year placement
Landeen, J et al	2013	Canada	Uniprofessional	31	Nursing students; Educators	Qualitative	Interviews; Focus groups	Problem-based learning
Leh, SK	2011	USA	Uniprofessional	42	Nursing students	Qualitative	Focus groups	Clinical rotation
Lemmon, ME et al	2018	USA	Uniprofessional	159	Medical students	Mixed methods	Questionnaire (open and closed questions); Observation data (electronic communication tracker); Focus groups	Clinical clerkship
Lewinson, L et al	2018	UK	Uniprofessional	13	Final-year nursing students	Qualitative	Interviews	N/A
Lingard, L et al	2003	Canada	Uniprofessional	21	Medical students	Qualitative	Interviews; Observation data (case presentations and related teaching exchanges)	Case presentations; Clinical clerkship
Lingard, L et al	2003	Canada	Uniprofessional	26	Medical students	Qualitative	Interviews; Observation data (case presentations and related teaching exchanges)	Case presentations; Clinical clerkship
Liou, KT et al	2019	USA	Uniprofessional	23	Medical students	Quantitative	Budner’s Tolerance of Ambiguity scale	Equine-facilitated learning
Llapa Rodrigues, EO et al	2016	Brazil	Uniprofessional	116	Nursing students	Quantitative	Questionnaire (KEZKAK Questionnaire, validated for the Portuguese language)	N/A
Lodewyk, K et al	2020	Canada	Uniprofessional	61	Medical students	Quantitative	Tolerance of Ambiguity in Medical Students and Doctors (TAMSAD); Questionnaire (investigator-developed sports background instrument)	N/A
Mangione, S et al	2018	USA	Uniprofessional	739	Medical students	Quantitative	Questionnaire (investigator-developed humanities exposure instrument; Brief Wisdom Screening Scale; Jefferson Scale of Empathy; Budner’s Tolerance for Ambiguity Scale; Wong and Law’s Emotional Intelligence Scale; 10-item generalized self-efficacy scale; Santa Barbara Solids Test	Humanities activities
Markey, K et al	2018	Ireland	Uniprofessional	30	Nursing students; Nurses	Qualitative	Interviews; Focus groups	N/A
Markey, K et al	2019	Ireland	Uniprofessional	71, 30	Nursing students; Nurses	Qualitative	Interviews; Focus groups	Clinical setting
Matchim, Y & Raetong, P	2018	Thailand	Uniprofessional	21	Nursing students	Qualitative	Interviews	Clinical setting
Maudsley, G et al	2008	UK	Uniprofessional	695	Medical students	Mixed methods	Questionnaire (open questions resulting in textual and numerical data)	Problem-based learning
McCarthy, J et al	2018	Ireland	Multiprofessional	12	Nursing students (Intellectual disability, mental health); Midwifery students	Qualitative	Interviews	Clinical placements
Merrill, JM et al	1994	USA	Uniprofessional	1009	Medical students	Quantitative	Questionnaire (investigator-developed intolerance of ambiguity instrument, incorporating Budner’s Tolerance for Ambiguity Scale)	N/A
Mol, SS et al	2019	Netherlands	Uniprofessional	35	Medical students; Doctors	Mixed methods	Questionnaire; Focus groups; Observation data (logbooks)	Clinical setting
Morton, KR et al	2000	USA	Uniprofessional	130	Medical students	Quantitative	Questionnaire (Budner’s Intolerance of Ambiguity, The Interpersonal Reactivity Index); Examination performance (standardized patient ratings)	N/A
Nevalainen, MK et al	2010	Finland	Uniprofessional	22	Medical students	Qualitative	Observation data (reflective learning diaries)	Reflective thinking course; Clinical setting
Nevalainen, MK et al	2012	Finland	Uniprofessional	307	Fifth-year medical students	Quantitative	Questionnaire (investigator-developed intolerance of uncertainty instrument)	Clinical setting
Neve, H et al	2017	UK	Uniprofessional	22	Medical students; Educators	Qualitative	Observation data (audio-diaries, discussion groups)	Small groups (‘Jigsaw’ groups)
Nguyen, M et al	2016	Canada	Uniprofessional	58	Medical students; Educators	Quantitative	Questionnaire (investigator-developed instrument with versions for students and educators, open and closed questions)	Arts-based learning activities
Nixon, J et al	2014	USA	Uniprofessional	191	Medical students	Quantitative	Observation data (educational prescriptions)	SNAPPS-Plus i.e. includes a PICO-formatted educational prescription; Case presentations; Clinical clerkships
Porteous, DJ & Machin, A	2018	UK	Uniprofessional	10	First-year nursing students (child, mental health, learning disability, adult)	Qualitative	Interviews; Observation data (audio-diaries)	N/A
Ramos-Morcillo, AJ et al	2020	Spain	Uniprofessional	32	Nursing students	Qualitative	Interviews	N/A
Riegelman, RK et al	1983	USA	Uniprofessional	198	Medical students	Quantitative	Questionnaire (Investigator-developed literature-reading instrument)	Reading medical literature
Rowan, CJ et al	2008	UK	Uniprofessional	96	Midwifery students	Qualitative	Focus groups	Problem-based learning
Sawanyawisuth, K et al	2015	Thailand	Uniprofessional	32	Fifth-year medical students	Quantitative	Observation data (audio-recorded case presentations)	SNAPPS; Case presentations; Clinical clerkship
Schéle, I et al	2011	Sweden	Uniprofessional	15	Dentistry students; Educators	Qualitative	Interviews	N/A
Scott, A et al	2020	UK	Uniprofessional	45	Medical students	Qualitative	Observation data (debriefing transcripts)	Simulation-based learning
Senette, L et al	2013	USA	Multiprofessional	26	Nursing students; Other students	Mixed methods	Questionnaire (incorporating Attitude Toward Collaborative Learning Scale, open and closed questions)	Simulation-based learning
Sobal, J & DeForge, BR	1991	USA	Uniprofessional	171	Medical students	Quantitative	Questionnaire (investigator-developed tolerance of uncertainty instrument, incorporating Budner’s Tolerance for Ambiguity Scale)	N/A
Stephens, GC et al	2020	Australia	Uniprofessional	207, 24	Medical students	Qualitative	Interviews; Observation data (online discussion forum text)	Online discussion of anatomy topics
Steinauer, JE et al	2018	USA	Uniprofessional	26	Fourth-year medical students	Qualitative	Interviews	Clinical setting
Stone, JP et al	2015	Canada	Uniprofessional	72	Final-year medical students; Graduated doctors	Mixed methods	Questionnaire (investigator-developed with open and closed questions)	Clinical setting
Toivonen, AK et al	2017	Finland	Uniprofessional	351	Fourth-year medical students	Qualitative	Observation data (written reflections)	Communication skills course
Vae, KJU et al	2018	Norway	Uniprofessional	33	Nursing students; Educators	Qualitative	Interviews	Clinical setting
Van Ryn, M et al	2014	USA	Uniprofessional	4732	First-year medical students	Quantitative	Questionnaire (incorporating Jefferson Empathy Scale Student Version, The Medical Authoritarianism Scale, and portions of Interpersonal Reactivity Index, Need for Closure Scale, Social Dominance Orientation Scale, Pearlin’s Mastery Scale, Rosenberg Self Esteem Scale, Patient-Reported Outcome Measurement Information System Short Forms Scales	N/A
Warner, TD et al	2001	USA	Uniprofessional	166	Medical students	Quantitative	Questionnaire (investigator-developed instrument)	N/A
Watkins, KD et al	2011	South Africa	Uniprofessional	44	Nursing students	Qualitative	Interviews; Focus Groups; Observation data (written diaries)	N/A
Wayne, S et al	2011	USA	Uniprofessional	313	Medical students	Quantitative	Questionnaire (Medical Students’ Attitudes Toward the Underserved, Budner’s Intolerance of Ambiguity Scale)	N/A
Weurlander, M et al	2019	Sweden	Uniprofessional	14	Medical students	Qualitative	Focus groups	N/A
Wolpaw, T et al	2009	USA	Uniprofessional	64	Medical students	Quantitative	Observation data (audio-recorded case presentations)	SNAPPS; Case presentations; Clinical clerkship
Wolpaw, T et al	2012	USA	Uniprofessional	60	Medical students	Qualitative	Secondary analysis of audio-recorded case presentations	SNAPPS; Case presentations; Clinical clerkship
Young-Brice, A et al	2018	USA	Uniprofessional	20	Nursing students	Qualitative	Interviews	N/A

N/A, not applicable; nc, not clear

**Table 3:** The ubiquity of uncertainty: a scoping review on how undergraduate health professions’ students engage with uncertainty: Summary of main findings

Theme	Sub-theme	Description	Studies
Learners’ interactions with uncertainty	Types of learners	A wide variety of health professions’ learners meet uncertainty within the context of their undergraduate studies. Most studies reported on cohorts of medical and nursing students, with mentions also of physiotherapy, midwifery, veterinary, dentistry and pharmacy students. All stages of undergraduate training are represented	Finnerty & Pope 2005; Rowan et al. 2008; Schéle et al. 2011; Nevalainen et al. 2012; Hazel et al. 2013; Hancock et al. 2017; Friary et al. 2018; Porteous and Machin 2018; Brondani & Donnelly 2020; Jowsey et al. 2020; Kashbour et al. 2019
	Types of uncertainty	Types of uncertainty can be categorised into: (i) uncertainty related to practice of healthcare itself; (ii) uncertainty related to the educational process; and (iii) uncertainty related to the self. The types of uncertainty that learners experienced, and their concerns around these, evolved as they progressed through their education	Sobal & DeForge 1991; Biley & Smith 1999; Huijer et al. 2000; Carr et al. 2001; Warner et al. 2001; Lingard et al. 2003a; Martinez & Lo 2008; Maudsley et al. 2008; Rowan et al. 2008; Ganesh & Ganesh 2010; Nevalainen et al. 2010; Krupat et al. 2011; Leh 2011; Schéle et al. 2011; Teunissen & Westerman 2011; Watkins et al. 2011; Nevalainen et al. 2012; Hazel et al. 2013; Landeen et al. 2013; Curtis 2014; Kristiansson et al. 2014; Nixon et al. 2014; Bassett et al. 2015; Ion et al. 2015; Stone et al. 2015; Ali et al. 2017; Toivonen et al. 2017; Dodgson et al. 2018; Handwerker 2018; Lewinson et al. 2018; Markey et al. 2018; Matchim & Raetong 2018; McCarthy et al. 2018; Porteous & Machin 2018; Vae et al. 2018; Young-Brice et al. 2018; Ingvarsson et al. 2019; Kashbour et al. 2019; Markey et al. 2019; Mol et al. 2019; Weurlander et al. 2019; Brondani and Donnelly 2020; Gonzalo et al. 2020; Koufidis et al. 2020; Ramos-Morcillo et al. 2020
Factors that influence learner experiences	Individual factors	There was some evidence that factors such as sex, age, background, discipline, and stage of training could impact on learner experiences of uncertainty, but the heterogeneity of study designs made it difficult to draw general conclusions	DeForge & Sobal 1989; Geller et al. 1990; Bingyou 1991; Sobal & DeForge 1991; Merrill et al. 1994; Nevalainen et al. 2010; Klugman et al. 2011; Evans et al. 2012; Landeen et al. 2013; Han et al. 2015; Eley et al. 2017; Hammond et al. 2017; Hancock et al. 2017; Young-Brice et al. 2018; Jowsey et al. 2020; Lodewyk et al. 2020
	System factors	Studies described a range of local and wider contextual factors which impacted on how learners experience uncertainty	Riegelman et al. 1983; Carr et al. 2001; Lingard et al. 2003a; Lingard et al. 2003b; Finnerty & Pope 2005; Frambach et al. 2012; Senette et al. 2013; Al-Kloub et al. 2014; Curtis 2014; Ion et al. 2015; Sawanyawisuth et al. 2015; Markey et al. 2018; McCarthy et al. 2018; Markey et al. 2019; Weurlander et al. 2019
Educational outcomes	Negative narrative	Overall, the narrative around learners’ experience of uncertainty tended to be negative. This was particularly evident for studies which described nursing students’ experiences in the clinical setting	Ganesh & Ganesh 2010; Krupat et al. 2011; Leh 2011; Watkins et al. 2011; Nevalainen et al. 2012; Stone et al. 2015; Llapa Rodrigues et al. 2016; Porteous and Machin 2018; Toivonen et al. 2017; Handwerker 2018; Dodgson et al. 2018; Helmich et al. 2018; Markey et al. 2018; McCarthy et al. 2018; Steinauer et al. 2018; Weurlander et al. 2019; Groot et al. 2020; Koufidis et al. 2020; Mol et al. 2019
	Learner approaches to uncertainty	Several papers indicated that an ability to manage uncertainty represented an important component of learners’ professional identity. Learners displayed a wide range of approaches to uncertainty. Some studies commented on learners avoiding or denying uncertainty, especially in situations where they were being assessed. Many researchers indicated that learners undergo a maturation process with respect to uncertainty	Riegelman et al. 1983; Geller et al. 1990; Merrill et al. 1994; Huijer et al. 2000; Lingard et al. 2003a; Lingard et al. 2003b; Balentine et al. 2010; Ganesh & Ganesh 2010; Nevalainen et al. 2010; Schéle et al. 2011; Frambach et al. 2012; Nevalainen et al. 2012; Landeen et al. 2013; Al-Kloub et al. 2014; Curtis 2014; Kristiansson et al. 2014; Han et al. 2015; Sawanyawisuth et al. 2015; Neve et al. 2017; Helmich et al. 2018; Mangione et al. 2018; Markey et al. 2018; Porteous & Machin 2018; Steinauer et al. 2018; Kashbour et al. 2019; Markey et al. 2019; Stephens et al. 2020
	Impact on learning	Studies examined correlations between students’ capacity to manage uncertainty in relation to their academic performance, career preferences, ability to empathise, and attitudes towards patients. Several papers proposed that uncertainty presents a barrier to learning, whilst others considered that it could be productive	Morton et al. 2000; Lingard et al. 2003a, b; Finnerty & Pope 2005; Ironside et al. 2009; Nevalainen et al. 2010; Wayne et al. 2011; Frambach et al. 2012; Al-Kloub et al. 2014; Duvivier et al. 2014; van Ryn et al. 2014; Eley et al. 2017; Friary et al. 2018; Mangione et al. 2018; Markey et al. 2018; McCarthy et al. 2018; Steinauer et al. 2018; Kashbour et al. 2019; Groot et al. 2020
Teaching and learning approaches		Several papers discussed specific “homes” within health professions’ curricula for supporting learning around uncertainty. Uncertainty was highlighted as a component of topics such as professionalism, communication, ethics, clinical reasoning, evidence-based medicine, and interprofessional learning. Direct teaching strategies included arts-based teaching, clinical case presentations using the SNAPPS model, Learning-by-Concordance, simulation and equine-facilitated learning. Other teaching strategies included: problem-based learning, clinical teaching, humanities teaching, simulation, team-based learning, small group learning, tactical games, online discussion of anatomy topics, and virtual patients. Reflection and reflective practice were also mentioned within the literature	Biley & Smith 1999; Ironside 2003; Lingard et al. 2003a, b; Finnerty & Pope 2005; Koh et al. 2008; Maudsley et al. 2008; Rowan et al. 2008; Wolpaw et al. 2009; Ganesh & Ganesh 2010; Nevalainen et al. 2010; Wayne et al. 2011; Curtis et al. 2012; Wolpaw et al. 2012; Hazel et al. 2013; Landeen et al. 2013; Gibson et al. 2014; Han et al. 2014; Kristiansson et al. 2014; Nixon et al. 2014; Chan et al. 2015; Han et al. 2015; Sawanyawisuth et al. 2015; Drummond et al. 2016; Fernandez et al. 2016; Gormley & Fenwick 2016; Hayward et al. 2016; Johnsen 2016; Nguyen et al. 2016; Ali et al. 2017; Bentwich & Gilbey 2017; Neve et al. 2017; Toivonen et al. 2017; Gaufberg et al. 2018; Gowda et al. 2018; Helmich et al. 2018; Lemmon et al. 2018; Mangione et al. 2018; Porteous & Machin 2018; Young-Brice et al. 2018; He et al. 2019; Kashbour et al. 2019; Liou et al. 2019; Weurlander et al. 2019; Brondani & Donnelly 2020; Fagundes et al. 2020; Gärtner et al. 2020; Groot et al. 2020; Jowsey et al. 2020; Lodewyk et al. 2020; Scott et al. 2020; Stephens et al. 2020

### Identified themes and sub-themes

Four major themes were identified: “Learners’ interactions with uncertainty”; “Factors that influence learner experiences”; “Educational outcomes”; and, “Teaching and learning approaches”.

### Learners’ interactions with uncertainty

#### Types of learners

A wide variety of health professions’ learners meet uncertainty within the context of their undergraduate studies. The vast majority of studies reported on cohorts of medical and nursing students; however, experiences of uncertainty were also recorded within midwifery, physiotherapy, veterinary, dentistry and pharmacy student cohorts (Finnerty and Pope 2005; Friary et al. 2018; Hancock et al. 2017; Hazel et al. 2013; Schéle et al. 2011; Rowan et al. 2008; Porteous and Machin 2018; Nevalainen et al. 2012; Kashbour et al. 2019; Brondani and Donnelly 2020; Jowsey et al. 2020). Studies included learners at all stages of their undergraduate training.

#### Types of uncertainty

Learners’ experiences of uncertainty, could be categorised as: (i) uncertainty related to the practice of healthcare itself (Ali et al. 2017; Nixon et al. 2014; Sobal and Deforge 1991; Carr et al. 2001; Lingard et al. 2003a; Ganesh and Ganesh 2010; Markey et al. 2018; Weurlander et al. 2019); (ii) uncertainty related to the educational process (Biley and Smith 1999; Dodgson et al. 2018; Mc Carthy et al. 2018; Hazel et al. 2013; Leh 2011; Stone et al. 2015; Maudsley et al. 2008; Gonzalo et al. 2020); and (iii) uncertainty related to the learner’s self (Ganesh and Ganesh 2010; Toivonen et al. 2017; Lingard et al. 2003a; Schéle et al. 2011; Vae et al. 2018; Young-Brice et al. 2018; Handwerker 2018; Nevalainen et al. 2010, 2012; Huijer et al. 2000; Weurlander et al. 2019). Uncertainty emerged when learners experienced differences between themselves and others (Ion et al. 2015; Watkins et al. 2011; Lewinson et al. 2018; Curtis 2014; Martinez and Lo 2008; Markey et al. 2019), unfamiliar situations, or issues lacking easily distinguishable solutions (Ion et al. 2015; Watkins et al. 2011; Lewinson et al. 2018; Matchim and Raetong 2018; Warner et al. 2001; Toivonen et al. 2017; Bassett et al. 2015). Common places where this happened were at transitions (e.g., entry into undergraduate studies, movement into, and between, clinical placements) (Porteous and Machin 2018; McCarthy et al., 2018; Ingvarsson et al. 2019; Teunissen and Westerman 2011), and in specific environments such as problem-based learning (Maudsley et al. 2008; Landeen et al. 2013; Rowan et al. 2008), and clinical settings (Krupat et al. 2011; Leh 2011; McCarthy et al. 2018; Kashbour et al. 2019; Mol et al. 2019; Koufidis et al. 2020). Several studies commented on how the types of uncertainty that learners experienced, and their concerns around these, evolved as they progressed through their education (Sobal and Deforge 1991; Kristiansson et al. 2014). Finally, the uncertainties faced by students in the context of the global coronavirus pandemic began to emerge in studies published in 2020 (Brondani and Donnelly 2020; Ramos-Morcillo et al. 2020).

### Factors that influence learner experiences

#### Individual factors

A large proportion of the literature examined individual learner differences with some evidence that gender, age, background, discipline, and stage of training could impact on how learners interact with uncertainty (Hancock et al. 2017; Bingyou 1991; Geller et al. 1990; Landeen et al. 2013; Nevalainen et al. 2010; DeForge and Sobal 1989; Eley et al. 2017; Young-Brice et al. 2018; Lodewyk et al. 2020; Jowsey et al. 2020). However, the heterogeneity of study designs made it difficult to draw general conclusions. For example, whilst some studies suggested that male students managed uncertainty better than female (Nevalainen et al. 2010), others suggested that females fared better (DeForge and Sobal 1989; Merrill et al. 1994; Geller et al. 1990); a further three papers found no gender differences (Sobal and Deforge 1991; Evans et al. 2012; Klugman et al. 2011). Several researchers commented on the multi-dimensional nature of uncertainty, and how different assessment instruments can lead to different outcomes (DeForge and Sobal 1989; Merrill et al. 1994; Hammond et al. 2017; P. K. J. Han et al. 2015).

#### System factors

Other studies identified a range of non-individual, or system, factors which influenced learners’ experiences of uncertainty. Studies identified both local (i.e., specific clinic setting, organisational culture) (Senette et al. 2013; Ion et al. 2015; Markey et al. 2018, 2019; Weurlander et al. 2019), and wider (i.e., professional socialisation, socio-cultural issues) (Curtis 2014; McCarthy et al. 2018; Finnerty and Pope 2005; Sawanyawisuth et al. 2015; Al-Kloub et al. 2014; Frambach et al. 2012; Weurlander et al. 2019) contextual factors that impacted on how learners experience uncertainty. Several papers described a health professions’ culture which, paradoxically, places value on certainty over uncertainty (Lingard et al. 2003a, 2003b; Riegelman et al. 1983).

### Educational outcomes

#### Negative narrative

Overall, the narrative around learners’ experience of uncertainty tended to be articulated in negative terms. Researchers described these experiences using words such as “discomfort”, “stress”, “anxiety”, and “vulnerability” (Handwerker 2018; Krupat et al. 2011; Leh 2011; Porteous and Machin 2018; McCarthy et al. 2018; Markey et al. 2018; Toivonen et al. 2017; Dodgson et al. 2018; Ganesh and Ganesh 2010; Helmich et al. 2018; Llapa Rodrigues et al. 2016; Nevalainen et al. 2012; Watkins et al. 2011; Steinauer et al. 2018; Stone et al. 2015; Weurlander et al. 2019; Mol et al. 2019; Groot et al. 2020; Koufidis et al. 2020). This was particularly evident for studies which described nursing students’ experiences in the clinical setting (Handwerker 2018; Porteous and Machin 2018; Llapa Rodrigues et al. 2016; Mc Carthy et al. 2018; Hazel et al. 2013; Leh 2011; Markey et al. 2018; Watkins et al. 2011; Leh 2011; Dodgson et al. 2018).

#### Learner approaches to uncertainty

Several papers indicated that an ability to manage uncertainty represented an important component of learners’ professional identity (Kristiansson et al. 2014; Mangione et al. 2018; Nevalainen et al. 2012; Neve et al. 2017). Learners themselves displayed a wide range of approaches to uncertainty (Nevalainen et al. 2012; Porteous and Machin 2018; Kristiansson et al. 2014; Markey et al. 2018, 2019; Helmich et al. 2018; Kashbour et al. 2019; Stephens et al. 2020). Strategies described in the literature included: learners letting go of perfectionism, adapting ideals to fit reality, being honest when lacking knowledge, asking for help, and understanding what it means to be “good enough”(Curtis 2014; Kristiansson et al. 2014; Schéle et al. 2011; Ganesh and Ganesh 2010; Nevalainen et al. 2012).

Learners tended to avoid or deny uncertainty, especially in assessment situations. Whilst some learners attempted to “self-preserve”, by avoiding expressions of uncertainty (Lingard et al. 2003a, b) and avoiding asking questions (Markey et al. 2018; Huijer et al. 2000), others appeared to place blame onto patients (Steinauer et al. 2018). This position was countered by one qualitative study, which found scant evidence of a denial of uncertainty in their medical student cohort (Kristiansson et al. 2014). Several papers highlighted the importance of socio-cultural background, e.g., country of origin, on learners’ likelihood to respond openly to uncertainty (Al-Kloub et al. 2014; Frambach et al. 2012; Sawanyawisuth et al. 2015).

Many researchers described a maturation process, i.e., that learners’ responses to uncertainty evolve as they accumulate experience and academic maturity (Kristiansson et al. 2014; Landeen et al. 2013; Nevalainen et al. 2010, 2012; Sobal and Deforge 1991; Merrill et al. 1994; Lingard et al. 2003b; Neve et al. 2017; Han et al. 2015; Riegelman et al. 1983; Balentine et al. 2010; Stephens et al. 2020). Only one study indicated that uncertainty tolerance did not change as learners progressed through their training, a finding which may relate to the study’s cross-sectional design (Geller et al. 1990).

#### Impact on learning

Several papers discussed the links between students’ capacity to manage uncertainty and their academic performance (Ironside et al. 2009; Morton et al. 2000; Groot et al. 2020), career preferences (Eley et al. 2017; Geller et al. 1990; Merrill et al. 1994; Nevalainen et al. 2010), ability to empathise (Markey et al. 2018; Mangione et al. 2018; Morton et al. 2000; van Ryn et al. 2014), and attitudes towards patients (Steinauer et al. 2018; Geller et al. 1990; Wayne et al. 2011; Merrill et al. 1994; Lingard et al. 2003b) with mixed and occasionally conflicting findings. Several papers proposed that uncertainty presents a barrier to learning, i.e., causing students to become less self-directed, proactive, and effortful in their learning (Al-Kloub et al. 2014; Frambach et al. 2012; Finnerty and Pope 2005; Duvivier et al. 2014). Other researchers commented that uncertainty under certain circumstances could be “productive”, i.e., where appropriate supports are in place, this can act as a catalyst for learning (Friary et al. 2018; McCarthy et al. 2018; Kashbour et al. 2019).

### Teaching and learning approaches

Several studies focused on existing approaches to teaching and learning around uncertainty from the perspectives of content (“what”) and process (“how”). With regards to the former, learners met uncertainty when engaging with topics such as professionalism, communication, ethics, clinical reasoning, evidence-based medicine, and inter-professional learning (Han et al. 2014, 2015; Hazel et al. 2013; Chan and Nyback 2015; Lemmon et al. 2018; Johnsen 2016; Ironside 2003; Jowsey et al. 2020). With regards to the latter, a number of formal teaching strategies which intended to help learners to work with uncertainty, were described. These studies largely fell into two groups: arts-based teaching which addressed issues of uncertainty and ambiguity (Klugman et al. 2011; Nguyen et al. 2016; Bentwich and Gilbey 2017; He et al. 2019), and clinical teaching which used SNAPPS, a clinical reasoning scaffold with a specific focus on identifying uncertainties (Nixon et al. 2014; Sawanyawisuth et al. 2015; Wolpaw et al. 2009, 2012; Fagundes et al. 2020). Other studies suggested that learners could develop ways to manage uncertainty through use of the Learning-by-Concordance approach (Fernandez et al. 2016), simulation (Scott et al. 2020) and a novel equine-facilitated workshop which introduced horses to medical students as “experiential surrogates for ambiguity” (Liou et al. 2019).

Learners also had opportunities to develop their capacity to manage uncertainty in other, more indirect ways, e.g., through problem-based learning (Maudsley et al. 2008; Rowan et al. 2008; Landeen et al. 2013; Koh et al. 2008) and simulation (Senette et al. 2013; Gormley and Fenwick 2016; Bintley et al. 2019; Gärtner et al. 2020; Groot et al. 2020; Jowsey et al. 2020). With regards to the former, researchers recommended that sessions should be actively tutored, and cases not overtly scripted, to support learning around uncertainty (Landeen et al. 2013; Biley and Smith 1999; Maudsley et al. 2008). Teaching in the clinical setting was also important, with an emphasis on building supportive educator-learner relationships (Lingard et al. 2003b; Finnerty and Pope 2005; Porteous and Machin 2018; Curtis et al. 2012).

Other educational strategies that emerged included team-based learning (Hazel et al. 2013), small group learning (Gibson et al. 2014; Chan and Nyback 2015), tactical games (Drummond et al. 2016), virtual patients (Hayward et al. 2016), online discussion of anatomy topics (Stephens et al. 2020), and non-specified humanistic activities (Mangione et al. 2018; Gaufberg et al. 2018). Reflective practice was also mentioned within the literature and researchers described a variety of techniques which could be usefully applied, including: discussions with mentors (Finnerty and Pope 2005; Kashbour et al. 2019), small group exercises (Neve et al. 2017; Ali et al. 2017), written reflection (Kristiansson et al. 2014; Ganesh and Ganesh 2010; Brondani and Donnelly 2020), and combinations of these (Nguyen et al. 2016; Chan and Nyback 2015; Gowda et al. 2018; Nevalainen et al. 2010; Toivonen et al. 2017; Kashbour et al. 2019).

Specific teaching approaches to support learning around uncertainty were mentioned within the studies. These included: helping learners to reach a sense of “good enough” (Kristiansson et al. 2014); encouraging learners to keep questioning what they think they know (Ali et al. 2017); directly acknowledging that ambiguity and uncertainty exist within health professions’ work (Wayne et al. 2011; Weurlander et al. 2019); helping learners to understand that success has different meanings; teaching thinking in ways that preserve uncertainty and fallibility (Ironside 2003); managing expectations around controlling uncertainty (Helmich et al. 2018); leveraging learners’ experiences of uncertainty in non-academic settings such as sports participation (Lodewyk et al. 2020), and providing extra support to ethnic minority students (Young-Brice et al. 2018). Table 3 shows a summary of our major findings.

## Discussion

In seeking to explore how undergraduate health professions' students learn to engage with uncertainty in their professional practice, this review highlights that the experience of uncertainty is ubiquitous within their education. It is clear that a wide variety of learners, from different professions and countries, engage with uncertainty at all stages of their training.

The review sheds light on the nuances of uncertainty for health professions’ learners. Different types exist; from the uncertainty related to interactions with the healthcare and educational processes, to the uncertainty students experience in relation to their own selves. These types of uncertainty arise for learners in many varied teaching and learning settings (although uncertainty related to lecture-based teaching was conspicuous in its absence). Problem-based learning seems to provide an important crucible for engaging with uncertainty, as does workplace-based learning. Our review also reinforces the idea that transitions, e.g., entering clinical rotations, provoke experiences of uncertainty for health professions’ learners (Teunissen and Westerman, 2011; Ingvarsson et al. 2019).

In keeping with the wider literature, this review highlights the various ways in which learners navigate uncertainty, and that both individual and context-related factors influence this process. It seems that learners also build a capacity to manage uncertainty as they progress through their training. Several studies refer to this phenomenon as a “maturation process”, and it’s unclear to what extent this unfolds due to students’ accumulation of learning and experience, or to socialisation within their chosen profession. Our findings lack detail around what, specifically, this maturation looks like. Existing longitudinal studies tend to track learners’ engagement with uncertainty through the lens of a psychological construct, i.e. tolerance of uncertainty (Hillen et al. 2017). However, cross-sectional qualitative studies suggest that the learners mobilise a wide range of knowledge, skills and attitudes in relation to uncertainty, a level of granular detail which may not be captured fully by existing research designs.

Whilst our review suggests that students meet with uncertainty many times during their training, there were few examples of direct teaching, i.e., through arts-based approaches (Klugman et al. 2011; Nguyen et al. 2016; Bentwich and Gilbey 2017; He et al. 2019) or clinical cases (Nixon et al. 2014; Sawanyawisuth et al. 2015; Wolpaw et al. 2009, 2012; Fernandez et al. 2016; Fagundes et al. 2020). When compared to other non-technical domains such as communication and team skills, this apparent scarcity is surprising (Buljac-Samardzic et al. 2010; Berkhof et al. 2011). This finding might be explained by how uncertainty and its management is conceptualised. Until recently, tolerance of uncertainty has largely been framed as a stable personality trait, although it is now considered at least partly amenable to training (Strout et al. 2018). The idea that the capacity to manage uncertainty is personality-driven, and is mostly taught indirectly rather than directly within health professions’ education, recalls the early days of the communication skills movement. Thirty years ago we asked ourselves “can communication skills be taught?”(Maguire 1990); could uncertainty management occupy a similar trajectory?

There may also be a reluctance to provide training around uncertainty due to its perception as a difficult, uncomfortable topic for healthcare professionals. Our review highlights a negative narrative around managing uncertainty, with learners’ frequently discussing it in terms of stress or strain. These descriptions link back to the wider literature which connects uncertainty with feelings of discomfort and anxiety (Carleton 2016; Shihata et al. 2016; Mishel 1984; Penrod 2001; Ilgen et al. 2018). In our review, this negativity was most apparent within cohorts of clinical nursing students. It is not clear whether there are particular characteristics to this context which are specifically negative, or if, perhaps, nursing students’ are more inclined to express and discuss the emotional aspects of their practice?

What this review does outline is that students’ experiences of uncertainty have several effects. In some cases, uncertainty acts as a barrier to learning (Al-Kloub et al. 2014; Frambach et al. 2012; Duvivier et al. 2014; Finnerty and Pope 2005; Scott et al. 2020). In others, it elicits behaviour change e.g., learners attempt to “self-preserve”, by avoiding expressions of uncertainty (Lingard et al. 2003a, 2003b) or even placing blame onto patients (Steinauer et al. 2018). This supports the idea that health professions’ learners feel pressure to preserve the semblance of competence in front of their teachers, engaging in impression management (Lo and Regehr 2017; McGaghie 2018; Patel et al. 2018).

The included studies say less on the benefits of engaging with uncertainty. One study (Friary et al. 2018) proposes that “some uncertainty or stress is needed to shift learning to a new level.” This is supported in the educational literature, where there is a growing recognition that experiences of uncertainty are important catalysts for deeper learning (Overoye and Storm 2015; Lodge et al. 2018). However, the authors highlight that uncertainty is only “productive” under certain circumstance i.e., when it does not undermine trust and confidence. It implies then that some experiences of uncertainty may be more helpful than others to students. This idea has been discussed previously with the idea that “good uncertainty… provides students opportunities to engage with the unknowns of a challenge in an otherwise supportive, well-structured environment”, whilst “bad” uncertainty can result in chaos (Beghetto 2017). In a health professions’ context we might hypothesise that a student who interacts with a patient from a different socio-cultural background, experiences a “productive” uncertainty, whilst one who can’t locate their classroom experiences one that is “unproductive”. There appears to be little objective data, and a gap in the literature, in relation to how these experiences are perceived and managed by students, and what outcomes result.

Despite the further issues that this review provokes around how learners engage with uncertainty, we do know that there are many opportunities for health professions’ educators to support them on this journey. Topics that commonly appear on health professions’ curricula, e.g., professionalism, communication, ethics, clinical reasoning, can provide a “home” for learning around uncertainty. Similarly, teaching settings such as problem-based learning contexts, and the clinical workplace lend themselves to experiential learning around this domain. Finally, educators can help their learners to manage and make sense of uncertain situations through supportive mentoring and role modelling, and through involving them in well-structured reflective exercises (Uygur et al. 2019).

### Future research

With regards to future research, an increased focus on longitudinal studies which employ qualitative or mixed method approaches could provide more detailed information on how students build their capacity to manage uncertainty during their training. Further work is also required to explore how learners’ experiences with specific types of uncertainty impact on learning processes, i.e., can we recognise and foster more “productive” experiences of uncertainty for students? Finally, expanding the scoping review approach to cover postgraduate training and cross-cultural studies, would improve our understanding of this issue.

### Strengths and limitations

We used a broad search strategy in order to maximise inclusivity and generate an overview of uncertainty in the literature. Thus we kept the initial search open to all levels of health professions’ training, an approach which yielded a high volume of papers. To limit the papers to a feasible data set, we chose to focus only on “uncertainty” and “ambiguity” (although we had tested other synonyms). Similarly, we restricted our searches to papers published during or after 1950, and to those published in the English language. Given the potential breadth of the field, future reviews may consider using variations of the search strategy we have documented, and might include utilising forward citation methods to improve the sensitivity and specificity of the literature search results.

## Conclusions

Training for uncertainty has been described as medical education’s “most elusive ideal”^28^. This scoping review allows us to track down this concern, providing an overview of how health professions’ students learn to engage with uncertainty during their undergraduate training. We have found that uncertainty is a ubiquitous concern in health professions’ education, with students experiencing different forms of uncertainty at many stages of their training. These experiences are influenced by both individual and system-related factors.

Whilst formal teaching to support learning around uncertainty is infrequent, specific strategies do exist, i.e., arts-based teaching, and clinical case presentations. Other types of teaching provide ways for students to meet with uncertainty indirectly, including problem-based learning, clinical teaching, humanities teaching, simulation, team-based learning, small group learning, tactical games, and virtual patients. Reflection and reflective practice are also mentioned as strategies to address learner experiences of uncertainty within the literature.

## Appendix 1

Preferred reporting items for systematic reviews and meta-analyses extension for scoping reviews (PRISMA-ScR) checklist.

**Table:**

Section	Item	PRISMA-ScR checklist Item	Reported on page #
Title			
Title	1	Identify the report as a scoping review	1
*Abstract*			
Structured summary	2	Provide a structured summary that includes (as applicable): background, objectives, eligibility criteria, sources of evidence, charting methods, results, and conclusions that relate to the review questions and objectives	1
*Introduction*			
Rationale	3	Describe the rationale for the review in the context of what is already known. Explain why the review questions/objectives lend themselves to a scoping review approach	3
Objectives	4	Provide an explicit statement of the questions and objectives being addressed with reference to their key elements (e.g., population or participants, concepts, and context) or other relevant key elements used to conceptualize the review questions and/or objectives	5
*Methods*			
Protocol and registration	5	Indicate whether a review protocol exists; state if and where it can be accessed (e.g., a Web address); and if available, provide registration information, including the registration number	n/a
Eligibility criteria	6	Specify characteristics of the sources of evidence used as eligibility criteria (e.g., years considered, language, and publication status), and provide a rationale	6
Information sources*	7	Describe all information sources in the search (e.g., databases with dates of coverage and contact with authors to identify additional sources), as well as the date the most recent search was executed	6
Search	8	Present the full electronic search strategy for at least 1 database, including any limits used, such that it could be repeated	Appendix 2
Selection of sources of evidence†	9	State the process for selecting sources of evidence (i.e., screening and eligibility) included in the scoping review	7
Data charting process‡	10	Describe the methods of charting data from the included sources of evidence (e.g., calibrated forms or forms that have been tested by the team before their use, and whether data charting was done independently or in duplicate) and any processes for obtaining and confirming data from investigators	7
Data items	11	List and define all variables for which data were sought and any assumptions and simplifications made	7
Critical appraisal of individual sources of evidence§	12	If done, provide a rationale for conducting a critical appraisal of included sources of evidence; describe the methods used and how this information was used in any data synthesis (if appropriate)	n/a
Synthesis of results	13	Describe the methods of handling and summarizing the data that were charted	7, 8
*Results*			
Selection of sources of evidence	14	Give numbers of sources of evidence screened, assessed for eligibility, and included in the review, with reasons for exclusions at each stage, ideally using a flow diagram	8
Characteristics of sources of evidence	15	For each source of evidence, present characteristics for which data were charted and provide the citations	Table 2
Critical appraisal within sources of evidence	16	If done, present data on critical appraisal of included sources of evidence (see item 12)	n/a
Results of individual sources of evidence	17	For each included source of evidence, present the relevant data that were charted that relate to the review questions and objectives	Table 2
Synthesis of results	18	Summarize and/or present the charting results as they relate to the review questions and objectives	Table 3
*Discussion*			
Summary of evidence	19	Summarize the main results (including an overview of concepts, themes, and types of evidence available), link to the review questions and objectives, and consider the relevance to key groups	15–19
Limitations	20	Discuss the limitations of the scoping review process	19
Conclusions	21	Provide a general interpretation of the results with respect to the review questions and objectives, as well as potential implications and/or next steps	20
Funding			
Funding	22	Describe sources of funding for the included sources of evidence, as well as sources of funding for the scoping review. Describe the role of the funders of the scoping review	21

*From:* Tricco AC, Lillie E, Zarin W, O'Brien KK, Colquhoun H, Levac D, et al. PRISMA Extension for Scoping Reviews (PRISMAScR): Checklist and Explanation. Ann Intern Med. 2018;169:467–473. https://doi.org/10.7326/M18-0850.

*JBI*, Joanna Briggs Institute; *PRISMA-ScR* , Preferred Reporting Items for Systematic reviews and Meta-Analyses extension for Scoping Reviews.

* where *sources of evidence* (see second footnote) are compiled from, such as bibliographic databases, social media platforms, and Web sites.

† A more inclusive/heterogeneous term used to account for the different types of evidence or data sources (e.g., quantitative and/or qualitative research, expert opinion, and policy documents) that may be eligible in a scoping review as opposed to only studies. This is not to be confused with *information sources* (see first footnote).

‡ The frameworks by Arksey and O’Malley (6) and Levac and colleagues (7) and the JBI guidance (4, 5) refer to the process of data extraction in a scoping review as data charting*.*

§ The process of systematically examining research evidence to assess its validity, results, and relevance before using it to inform a decision. This term is used for items 12 and 19 instead of "risk of bias" (which is more applicable to systematic reviews of interventions) to include and acknowledge the various sources of evidence that may be used in a scoping review (e.g., quantitative and/or qualitative research, expert opinion, and policy document).

## Appendix 2

The ubiquity of uncertainty: a scoping review on how undergraduate health professions’ students engage with uncertainty: search strategy used for MEDLINE.

**Table:**

PubMed	
1	(education[Title/Abstract]) OR educational[Title/Abstract] OR learning[Title/Abstract] OR "Learning"[Mesh] OR "Social Learning"[Mesh] OR “Education, Professional"[Mesh] OR "Education, Pharmacy, Graduate"[Mesh] OR "Education, Pharmacy"[Mesh] OR "Education, Nursing, Graduate"[Mesh] OR "Education, Nursing, Diploma Programs"[Mesh] OR "Education, Nursing, Continuing"[Mesh] OR "Education, Nursing, Baccalaureate"[Mesh] OR "Education, Nursing, Associate"[Mesh] OR "Education, Nursing"[Mesh] OR "Education, Medical, Undergraduate"[Mesh] OR "Education, Medical, Graduate"[Mesh] OR "Education, Medical, Continuing"[Mesh] OR "Education, Medical"[Mesh] OR "Education, Graduate"[Mesh] OR "Education, Dental, Graduate"[Mesh] OR "Education, Dental, Continuing"[Mesh] OR "Education, Dental"[Mesh] OR “Educational Measurement”[Mesh])
2	((student[Title/Abstract] or students[Title/Abstract]) AND (medical[Title/Abstract]] OR medicine[Title/Abstract] OR nursing[Title/Abstract] OR midwifery[Title/Abstract] OR midwives[Title/Abstract] OR pharmacy[Title/Abstract] OR pharmacist[Title/Abstract] OR physiotherapist[Title/Abstract) OR physiotherapists[Title/Abstract] OR dentist[Title/Abstract] OR dentists[Title/Abstract] OR veterinary[Title/Abstract] OR dental[Title/Abstract] OR "Students, Premedical"[Mesh] OR "Students, Pharmacy"[Mesh] OR "Students, Nursing"[Mesh] OR "Students, Medical"[Mesh] OR "Students, Health Occupations"[Mesh] OR "Students, Dental"[Mesh])
3	(uncertainty[Title/Abstract] or ambiguity[Title/Abstract])
4	1 AND 2 AND 3

## Appendix 3

The ubiquity of uncertainty: a scoping review on how undergraduate health professions’ students engage with uncertainty: key health professions’ journals that were hand-searched during this review.

**Table:**

Hand searched health professions’ journals
Academic Emergency Medicine
Academic Medicine Advances in Health Sciences Education BMC Medical Education Health Professions Education International Journal of Medical Education International Journal of Nursing Studies Journal of Dental Education Journal of Veterinary Medical Education Medical Education Medical Teacher Midwifery Möbius: A Journal for Continuing Education Professionals in Health Sciences The Clinical Teacher
